# Convergent validation of the Involuntary Autobiographical Memory Inventory across levels of analysis in a Polish sample

**DOI:** 10.1038/s41598-026-40606-3

**Published:** 2026-05-19

**Authors:** Krystian Barzykowski, Ewa Ilczuk, Sezin Öner, Paulina Chwiłka, Michał Wereszczyński, Wiktoria Jakubowska, Justyna Hobot, Michał Wierzchoń

**Affiliations:** 1 Applied Memory Research Laboratory, Institute of Psychology, Faculty of Philosophy, Jagiellonian University, Kraków, Poland; 2 Department of Psychology, Kadir Has University, Istanbul, Turkey; 3 Consciousness Lab, Institute of Psychology, Jagiellonian University, Krakow, Poland; 4 Centre for Brain Research, Jagiellonian University, Krakow, Poland

**Keywords:** Involuntary thoughts, Involuntary autobiographical memories, Involuntary future thoughts, Mind-wandering, Emotional distress, Interoception, Neuroscience, Psychology, Psychology

## Abstract

We adapted the Involuntary Autobiographical Memory Inventory (IAMI) into Polish and examined its psychometric properties and convergent validity using a multi-level validation approach. The IAMI assesses individual differences in the frequency of involuntary autobiographical memories and future-oriented thoughts. In Study 1, we conducted confirmatory factor analysis and tested convergent validity using a laboratory vigilance task that objectively captured involuntary past and future thoughts under controlled conditions. In Study 2, we replicated the factorial structure and further examined convergent validity through associations with mind-wandering, emotional distress, and interoceptive sensitivity. Across both studies, confirmatory factor analyses supported a two-factor structure distinguishing involuntary past and future thoughts, with strong internal consistency. Importantly, IAMI scores predicted both laboratory-elicited involuntary thoughts and theoretically related self-report constructs, providing convergent validity across self-report, behavioural, and bodily-level measures. Together, these findings demonstrate that the Polish adaptation of the IAMI is not only a reliable and valid instrument but also offers novel insights into the cognitive and embodied mechanisms underlying involuntary mental time travel. We discuss the implications of these findings for future research on spontaneous thought processes and their links to cognition and emotion.

**Supplementary Information:**

The online version contains supplementary material available at 10.1038/s41598-026-40606-3.

## Introduction

Intrusive thoughts may manifest as involuntary autobiographical memories (IAMs) or involuntary future thoughts (IFTs). Autobiographical remembering allows individuals to mentally travel through time, retrieving past experiences and imagining future events^1^. Although autobiographical memory research has prioritized voluntary autobiographical memory, i.e., focusing on memories that are intentionally recalled through deliberate cognitive effort, a substantial portion of autobiographical remembering occurs involuntarily, without any conscious intention or retrieval attempt. Involuntary autobiographical memories are spontaneously retrieved personal past events that enter awareness without a deliberate search process. Similarly, involuntary future thoughts (IFTs) involve the spontaneous emergence of mental images or scenarios of possible future events that individuals think of without intentional effort^2,3^, however, unlike IAMs they require to a higher extent simulation, not retrieval^4^.

Both IAMs and IFTs share phenomenological similarities, including vivid sensory detail, emotional intensity, and episodic specificity, though the extent to which these features are experienced may vary across individuals. Their spontaneous retrieval is highly cue-dependent, typically triggered by external stimuli (e.g., sights, sounds, or smells) or internal states (e.g., emotions, ongoing concerns, or goals). This interplay between memory retrieval and contextual or internal triggers underscores the context-sensitive nature of involuntary mental time travel, suggesting that IAMs and IFTs as one of the key cognitive processes that support everyday thought and experience.

### Prevalence and functional significance in everyday life of IAMs and IFTs

Previous research suggests that IAMs are as frequent as voluntary autobiographical memories^5–7^. IAMs commonly occur during moments of low cognitive engagement, such as when doing routine activities like driving a car^2,3,6,8^. In these contexts, attention is diffused rather than highly focused, allowing associative memory networks to be more readily activated by internal and external cues^9–12^. Also IFTs emerge when attentional resources are not fully engaged and environmental cues or current concerns prompt projections about upcoming events.

Involuntary thoughts may serve important functional and adaptive roles by facilitating emotional regulation, problem-solving, and future planning (but see^13,14^ publications, which argue for their non-functional and disruptive nature; or^15^ for a more nuanced view). They may help individuals navigate their environments effectively by spontaneously reminding them of past experiences that might be relevant to current or future situations^16,17^. Such spontaneous retrieval can contribute significantly to maintaining personal identity and guiding behaviour in adaptive ways^18^. Regardless of the ongoing debate about whether IAMs and IFTs may serve a functional purpose, it is evident that they occur frequently in everyday life and, depending on the context (e.g., valence, meta-awareness, goal congruence), can be either beneficial or detrimental^15^.

The frequency and emotional impact of involuntary thoughts may be influenced by situational relevance and personal significance. Research suggests that involuntary thoughts, among healthy individuals, are predominantly positive, mirroring findings from voluntary memory research^19^. However, in clinical contexts, IAMs can take on a distressing form, as observed in posttraumatic stress disorder (PTSD), in which intrusive, distressing memories frequently disrupt cognitive and emotional functioning^20,21^. Understanding the mechanisms underlying IAMs retrieval is thus essential for both general memory research and clinical applications. Also, IFTs can contribute to psychopathology. In PTSD, IFTs are often over-general or bleak, reinforcing avoidance and a sense of foreshortened future that impairs goal pursuit, while in generalized anxiety, they intrude as threatening scenarios that intensify hyper-arousal and avoidance behavior (e.g.,^22,23^). In depressive disorders, negative IFTs can be experienced as uncontrollable and can maintain ruminative, self-deprecating thought cycles, prolonging low mood. Overall, higher frequencies of intrusive thoughts are consistently linked to greater emotional distress across clinical populations (e.g.,^24,25^).

### Characteristics and determinants of involuntary thoughts

A core feature of involuntary thoughts is their cue-dependent nature. Unlike voluntary memories, which require a goal-directed search, IAMs arise when internal cognitive states or retrieval cues in the environment activate stored memory traces (e.g.,^26,27^). Studies have shown that involuntary thoughts are most frequently triggered by external stimuli that match specific features of the original encoding context^28^. This cue–memory match principle ensures that involuntary retrieval is selective in that it picks up memories with strong associations with present contextual elements^29^.

Beyond cue dependence, cognitive load significantly impacts the frequency of involuntary thoughts in that these thoughts emerge most frequently in states of low cognitive engagement, when executive resources are not fully allocated to a demanding task. Previous research using experimental tasks has well documented that involuntary thoughts are more common under low attentional load and low cognitive demands^12,30–34^. Similarly, verbal and pictorial cues presented alongside a cognitively demanding task elicit fewer IAMs than when attention is more diffused^35^. These findings highlight that executive control mechanisms have a gating role in the accessibility of involuntary thoughts.

### Individual differences in the frequency of involuntary thoughts

While involuntary thoughts seem to be universal and frequent in everyday life, individuals vary widely in how often they experience them. For example, diary studies tracking IAMs in naturalistic settings indicate substantial individual variability, with some individuals reporting frequent involuntary retrieval, while others experience IAMs only rarely^2,37,38^. This variability extends beyond frequency, showing differences in content, emotional tone, and functional impact. Some individuals tend to recall highly emotional or personally meaningful involuntary memories, whereas others experience more neutral or random involuntary memories^39^. Personality factors are linked with the frequency of involuntary thoughts (including mind wandering^40,41^. For example, while individuals high in openness to experience report more frequent IAMs, individuals with high conscientiousness and agreeableness report lower IAMs frequency, possibly suggesting that individuals with more structured cognitive styles (a way of thinking characterized by a preference for order, organization, clear rules, and systematic problem-solving) have greater control over spontaneous thoughts^6^.

Also, a range of cognitive and emotional factors may contribute to individual differences in IAMs frequency. One key factor is attentional control, suggesting that individuals with lower attentional control are more prone to task-unrelated thoughts, making them more susceptible to IAMs^31,36^. Similarly, individuals with lower working memory capacity experience more frequent IAMs and make greater efforts to avoid them, likely due to reduced control over intrusive thoughts and difficulty in suppressing unwanted recollections^42^. These findings align with evidence that reduced executive control accompanies the retrieval of IAMs, particularly in low cognitive load conditions^9^. Individuals with weaker attentional filtering mechanisms may experience more spontaneous involuntary thoughts, increasing their likelihood of IAMs.

Another relevant factor is emotional distress. For example, individuals with anxiety disorders tend to experience more involuntary thoughts, usually in the form of intrusions, and they perceive these thoughts as extremely distressing. Visual intrusions are often recurrent, difficult to control, and emotionally distressing^43^. Negative intrusions are also frequently reported in individuals with depression, who, compared to healthy controls, tend to experience more internally oriented and self-focused spontaneous thoughts. These thoughts are more often related to negative rather than positive content, and less externally oriented.)^44^. Even in the case of dysphoria, while the frequency of involuntary thoughts was not different from healthy controls, individuals with dysphoria perceived these IAMs more negatively^45^. Even in studies with nonclinical samples, IAMs frequency is positively correlated with measures of anxiety and rumination^24^. Overall, these findings suggest that IAMs are not simply a function of memory accessibility, but individual differences in affective regulation and stress reactivity influence involuntary retrieval likelihood.

Individuals high in trait anxiety, worry and neuroticism tend to experience more frequent intrusive thoughts, which are often more negatively valenced and emotionally intense^46^. This effect is likely mediated by heightened meta-awareness and increased attention to internal psychological states^47^. Thought suppression further complicates this picture, demonstrating a paradoxical relationship with intrusive thoughts. Efforts to suppress unwanted thoughts reliably predict higher frequencies of intrusive thoughts^48^. Contrary to theoretical expectations, cognitive control mechanisms, particularly inhibitory control, appear to play a limited role in determining intrusive thoughts frequency. Instead, factors such as cue sensitivity and working memory capacity seem more influential in determining when such thoughts occur. Additionally, intrusive thoughts frequency is positively associated with spontaneous mind-wandering and daydreaming propensity. Positively valenced daydreaming styles are more strongly related to future-oriented than to past-oriented involuntary thoughts, which suggests domain-specific patterns within spontaneous cognition^48^. Crucially, it is not the frequency of intrusive thoughts per se that causes dysfunctions, but content, emotional valence, control appraisal, and context.

Collectively, these findings indicate that both IAMs and IFTs frequency arises from a complex interplay of emotional dispositions, cognitive capacities, and regulatory strategies. This offers important insights into both normative variations in future-oriented thinking and potential vulnerabilities in clinical populations where dysregulated future cognition may contribute to psychological distress.

### Bridging the gap: toward a systematic Understanding of individual differences

Although IAMs and IFTs have been extensively studied in terms of their retrieval mechanisms and phenomenology^11,32,37,49^, research on individual differences in the frequency of involuntary thoughts remains limited. Addressing this gap, Berntsen et al.^24^ developed the Involuntary Autobiographical Memory Inventory (IAMI) as a standardized measure that characterizes individual differences in the frequency of involuntary thoughts. The tool consists of two correlated subscales assessing involuntary autobiographical memories and involuntary future thoughts. In addition to having strong psychometric properties, the discriminant validity of the scale was established with daydreaming and dissociation, showing the inventory’s unique capacity to measure involuntary thoughts. In line with previous evidence, the frequency of involuntary thoughts was found to correlate with emotional distress, including rumination, worry, PTSD symptoms, depression, and anxiety. Overall, IAMI provides a valuable tool for assessing the prevalence of involuntary autobiographical memories and future thoughts, offering insights into their role in emotional well-being and cognitive functioning.

However, to the best of the authors’ knowledge—and somewhat to our own surprise—the tool has so far been available only in English (i.e., no other language-specific psychometric adaptations exist), and therefore its applicability to non-English-speaking populations has remained limited. Developing versions in other languages could also open new avenues of research by examining whether language and culture—particularly in languages that rely more heavily on imagery-related terms (e.g.,^50^)—may influence the nature of involuntary past and future thoughts. Therefore, there is a need for proper psychometric adaptation and validation of IAMI in other languages to facilitate research on IAMs and IFTs among individuals from more diverse cultural and linguistic backgrounds, and to support broader cross-cultural investigations by researchers worldwide. Specifically, adapting the IAMI to the Polish language and cultural context can enable accurate and reliable assessment of past and future involuntary thoughts within Polish-speaking populations, ensuring linguistic and conceptual equivalence. This will allow for valid cross-cultural comparisons and contribute to the global generalizability of findings in memory research. Furthermore, it will provide a valuable tool for use in clinical, cognitive, and developmental studies conducted in Poland, enhancing the methodological rigor and relevance of psychological research in this context.

Beyond providing a language-specific adaptation of the IAMI, the present work also creates a unique opportunity to address theoretically important questions concerning the cognitive processing of spontaneous past and future thoughts. In this sense, the primary aim of the present study is not limited to psychometric validation per se, but rather to offer a cognitive–experimental contribution to the study of involuntary mental time travel. The Polish adaptation of the IAMI thus serves as a methodological vehicle rather than the main outcome of the investigation. Crucially, the present study extends prior validation efforts by examining the convergent validity of the IAMI using laboratory-based indices of involuntary cognition. This multi-level approach allows us to test convergence across self-report measures (IAMI scores), behaviourally derived indicators of spontaneous cognition (probe-caught involuntary past and future thoughts), and individual differences in bodily awareness (interoceptive sensitivity).

### The present research

In two studies, we adapted the Involuntary Autobiographical Memory Inventory (IAMI) into Polish and evaluated its psychometric properties, ensuring close linguistic and conceptual equivalence with the original version. Importantly, beyond providing a language adaptation, the present research was designed to offer a broader empirical contribution by examining the convergent validity of the IAMI across multiple, theoretically relevant levels of analysis. In Study 1, we assessed the factor structure and reliability of the Polish IAMI and tested its validity using laboratory-based measures of involuntary past and future thoughts obtained under highly controlled conditions using a probe-caught vigilance paradigm. This allowed us to examine whether individual differences captured by the questionnaire correspond to objectively measured spontaneous cognition. In Study 2, we further extended this validation by examining associations between IAMI scores and related constructs assessed via self-report, including mind-wandering, emotional distress, and interoceptive sensitivity—variables theoretically linked to involuntary thought processes. By integrating questionnaire data with behavioral and bodily-level indices of involuntary cognition, the present work goes beyond a standard psychometric adaptation and provides experimental evidence relevant to the cognitive mechanisms underlying involuntary mental time travel. To our knowledge, this is the first study to validate the IAMI using both subjective self-reports and objective laboratory measures, thereby strengthening its theoretical and methodological foundations. Neither the studies nor the hypotheses were preregistered.

## Study 1

### Method

Informed consent was obtained prior to data collection from all subjects and/or their legal guardian(s). Participants were informed that they were free to withdraw from the study at any point. The procedure was approved by the Jagiellonian University Research Ethics Committee (no: KE/39_2021). All methods and procedures were performed in accordance with the relevant institutional and international ethical guidelines and regulations.

### Participants

A total of 222 healthy Polish participants (55 males, 163 females, and 4 participants declared other gender) took part in the study. Ages of participants in the sample ranged from 18 to 56 years (*M* = 22.15, *SD* = 3.93). Participants were recruited online and received a financial reward for their participation in the study.

### Materials

#### The Polish adaptation of the IAMI

Permission to adapt the scale was granted by the first author of the original paper (D.B.). To develop the Polish version of IAMI, the scale was first translated from English into Polish by the authors (K.B., E.I.), and the first Polish version was agreed upon through discussion after comparing two independent translations. It was then back-translated into English with support of machine translation (i.e., DeepL Translator), and the wording was carefully checked and refined by a human translator (K.B.). Importantly, the translation process was primarily carried out by human translators, with only this single stage (i.e., back-translation) being supported by machine translation, and at no stage was the process conducted automatically without human supervision. The back-translation was subsequently reviewed in consultation with the first author of the original scale (D.B.), who provided comments and suggestions for adjustments. Based on the feedback, further revisions were independently made by two researchers (K.B., E.I.), and any disagreements were resolved through discussion. We therefore feel confident that the translation achieved close conceptual and linguistic equivalence to the original version. The final Polish version of IAMI is provided in the Supplementary Material/Appendix.

#### Vigilance task for measuring frequency of IAMs and IFTs in the laboratory

We employed a computerized vigilance task adapted from Schlagman and Kvavilashvili^32^, a paradigm used in several previous studies that is widely recognized as reliable and valid for studying involuntary thoughts in laboratory settings (e.g.,^12,31,34,51^). In brief, participants were presented with a continuous stream of slides and asked to identify rare target stimuli (vertical line patterns) among frequent non-target slides (horizontal lines). Some slides also featured verbal cues (e.g., “*driving a car*”) intended to trigger spontaneous thoughts. Depending on the research condition, some participants also completed an additional low-engagement 1-back task. However, given the extremely low frequency of target stimuli, the overall procedure remained simple and minimally demanding. Throughout the task, participants were probed 23 times at random intervals to record the thoughts they were experiencing at that moment. These thoughts were categorised both by participants (during the laboratory session) and later by trained judges as a part of data processing. As a result, thoughts were classified as task-related or task-unrelated, involuntary or voluntary, and according to their temporal orientation. The thought classification process was carried out under the guidelines outlined in the research protocol for collecting and coding involuntary past and future thoughts, which is described in detail by Barzykowski, Ilczuk, and Kvavilashvili^52^. Briefly, the first coding stage distinguished task-related thoughts (directly tied to the task, e.g., *I pressed the red button*), task-related interference (appraisals or reflections on the task, e.g., *I was wondering how many different patterns of lines are being used*), and task-unrelated thoughts (no relationship to the task, e.g., *memories of past events or plans for the future*). During the vigilance task, participants indicated whether each reported thought occurred deliberately (they chose to think about it) or involuntarily (it simply popped into their mind). Thoughts were then automatically classified as deliberate or involuntary based on participants’ responses, with only involuntary task-unrelated thoughts included in subsequent analyses. Crucially, the use of the vigilance task enabled a direct test of convergent validity by examining whether individual differences captured by the IAMI predict the occurrence of involuntary autobiographical memories and future-oriented thoughts under tightly controlled laboratory conditions.

### Procedure

Participants took part in a laboratory study during which they completed a computerized vigilance task (briefly described above). Upon completion of this task, participants remained at the workstation and filled out the IAMI questionnaire directly on the computers.

### Data analytics plan

We conducted confirmatory factor analysis (CFA) using jamovi^53^ to evaluate the hypothesized two-factor structure of the questionnaire, which distinguishes between past and future involuntary thoughts. This structure was derived from prior theoretical and empirical work^24^. The CFA was carried out using maximum likelihood estimation. To assess and potentially improve the model fit, we examined model modification indices and considered theoretically justified adjustments where appropriate, such as correlated residuals.

Model fit was assessed using multiple goodness-of-fit indices. The Chi-square statistic (χ²) was used to compare the observed and expected covariance matrices, where a non-significant value suggests a good model fit. However, due to its sensitivity to large sample sizes, this statistic was interpreted alongside other indices. The comparative fit index (CFI) and the Tucker-Lewis Index (TLI) were also examined, with values of 0.90 or higher indicating an acceptable fit. The root mean square error of approximation (RMSEA) was used to assess model misfit, with values of 0.08 or lower considered adequate, and values of 0.05 or lower considered good. Finally, the standardized root mean square residual (SRMR) was examined, with values of 0.08 or lower indicating an adequate fit.

After confirming the factorial structure of IAMI, we assessed the internal consistency of the scale. Finally, to evaluate its theoretical validity, we conducted correlational analyses examining the relationship between IAMI scores and the frequency of involuntary thoughts reported during the laboratory task.

## Results

### Data screening

Initially we screened the data for outliers and conducted normality checks for the study variables. Next, we examined the validity of responses (i.e., correct responses) in the laboratory vigilance task. The IAMI indices showed minimal departures from normality: Future IAMI (skew = − 0.22, SE = 0.16; kurtosis = − 0.36, SE = 0.33), Past IAMI (skew = − 0.12, SE = 0.16; kurtosis = − 0.37, SE = 0.33), and Total IAMI (skew = − 0.13, SE = 0.16; kurtosis = − 0.32, SE = 0.33). Shapiro–Wilk tests were non-significant for these variables, Ws = 0.99, ps = 0.106–0.435, indicating approximate normality. By contrast, the raw frequency variables (IAMs, IFTs) showed positive skew (skew = 0.84 and 1.11; SEs = 0.16) and modest kurtosis (0.30 and 1.08; SEs = 0.33), with Shapiro–Wilk tests indicating deviations from normality (Ws = 0.93 and 0.90, ps < 0.001). Because our primary analyses were based on bivariate correlations, which are robust to moderate non-normality in the absence of influential outliers, we retained all cases; no observations were excluded on the basis of normality. For the laboratory vigilance task (IAMs and IFTs), no invalid responses were detected.

### Descriptive statistics and internal consistency reliability

Although the IAMI was originally designed as a single-factor scale, model comparisons of its factor structure favoured a two-factor model, with correlated dimensions reflecting reports of involuntary past and future thoughts^24^. For that reason, scale statistics and further confirmatory factor analyses were reported based on this focal model. The full scale with 20 items demonstrated high reliability, with Cronbach’s alpha of 0.94. The past and future involuntary thoughts subscales also showed high internal consistency reliability, with Cronbach’s alphas of 0.90 and 0.89, respectively. Past items were rated higher than future items, *t*(221) = 4.75, *p* <.001, *M*_*diff*_ = 0.18, *SE* = 0.04, Cohen’s *d* = 0.32.

### Confirmatory factor analysis

The CFA model was estimated to test the hypothesized two-factor structure of IAMI (i.e., comprising two subscales: past and future involuntary thoughts). Covariances among the ten items within each subscale were freely estimated. As presented in Table 1, the model demonstrated acceptable fit to the data: χ²(158, *N* = 222) = 373, *p* <.001; CFI = 0.92; TLI = 0.90; RMSEA = 0.08, with a 90% confidence interval of 0.07 to 0.09, and SRMR = 0.06. Standardized factor loadings, presented in Table 2, ranged from 0.57 to 0.78, and all were statistically significant (*p* <.001), indicating that each item significantly loaded onto its respective latent factor. Consistent with the original IAMI, the correlation between the past and future involuntary thought subscales was high, *r*(220) = 0.76, *p* <.001 (Table 3).

**Table 1:** Model fit indices for confirmatory fit indices obtained in study 1&2.

	χ ^2^	df	CFI	TLI	SRMR	RMSEA
Study 1	373	158	0.92	0.90	0.06	0.08
Study 2	345	158	0.94	0.92	0.05	0.06

**Table 2:** Item-factor loadings in CFA (Study 1 & study 2).

	Items	Labels	Study 1	Study 2
Past	Q1_2	Memories of personal events pop into my mind by themselves—without me consciously trying to remember them.	0.710	0.732
	Q1_4	After something surprising has happened, I spontaneously remember it, without consciously trying. It just comes to me.	0.622	0.596
	Q1_8	After I have experienced something that made a strong impression, I spontaneously remember it, without consciously trying. It just comes to me.	0.592	0.583
	Q1_9	Some emotions, moods or thoughts bring memories of past events to mind—without me consciously trying to remember them.	0.727	0.746
	Q1_11	Listening to some music or songs bring memories of past events to mind—without me consciously trying to remember them.	0.635	0.661
	Q1_13	When I am relaxing or doing routine work, memories of past events come to my mind by themselves—without me consciously trying to remember them.	0.781	0.735
	Q1_14	When I am bored, memories of past events come to my mind by themselves—without me consciously trying to remember them.	0.783	0.764
	Q1_17	When I am physically active, for example walking, bicycling, or running, memories of past events come to my mind by themselves– without me consciously trying to remember them.	0.708	0.628
	Q1_19	Some locations or places bring memories of past events to mind—without me consciously trying to remember them.	0.727	0.700
	Q1_20	Some sensory experiences, such as some odors or tastes, bring memories of past events to mind—without me consciously trying to remember them.	0.601	0.653
Future	Q1_1	When I am relaxing or doing routine work, imaginary future events come to my mind by themselves—without me consciously trying to evoke them.	0.669	0.668
	Q1_3	Some locations or places bring imaginary future events to mind—without me consciously trying to evoke them.	0.731	0.605
	Q1_5	Imaginary future events pop into my mind by themselves—without me consciously trying to evoke them.	0.665	0.668
	Q1_6	Some emotions, moods or thoughts bring imaginary future events to mind—without me consciously trying to evoke them.	0.720	0.754
	Q1_7	When I am bored, imaginary future events come to my mind by themselves —without me consciously trying to evoke them.	0.691	0.694
	Q1_10	When I am physically active, for example walking, bicycling, or running, imaginary future event come to my mind by themselves— without me consciously trying to evoke them.	0.688	0.576
	Q1_12	After something surprising has happened, I spontaneously imagine related events in the future, without consciously trying. It just comes to me.	0.667	0.599
	Q1_15	Some sensory experiences, such as certain odors or tastes, bring imaginary future events to mind—without me consciously trying to evoke them.	0.573	0.542
	Q1_16	After I have experienced something that made a strong impression, I spontaneously imagine related events in the future, without consciously trying. It just comes to me.	0.707	0.574
	Q1_18	Listening to some music or songs bring imaginary future events to mind—without me consciously trying to evoke them.	0.631	0.588

**Table 3:** Pearson correlations between IAMI scores and laboratory-elicited involuntary autobiographical memories (IAMs) and future thoughts (IFTs).

	Total_IAMI		Future_IAMI		Past_IAMI		TotalIT		IAMs		IFTs	
Total_IAMI	—											
Future_IAMI	0.94	***	—									
Past_IAMI	0.93	***	0.76	***	—							
Laboratory based total number of involuntary thoughts	0.21	**	0.16	*	0.24	***	—					
Laboratory based number of IAMs	0.15	*	0.09		0.20	**	0.73	***	—			
Laboratory based number of IFTs	0.06		0.07		0.05		0.45	***	0.05		—	

Note. * *p* <.05, ** *p* <.01, *** *p* <.001. IAMI = Involuntary Autobiographical Memory Inventory; IAMs = involuntary autobiographical memories; IFTs = involuntary future thoughts.

### Intercorrelations with laboratory reports of involuntary thoughts

The types of involuntary thoughts reported in the laboratory setting significantly differed, F (2,440) = 17.10, *p* <.001, η_p_^2^ = 0.07. Participants reported more IAMs than IFTs, *t*(220) = 4.02, *p* <.001, *M*_*diff*_ = 1.14, *SE* = 0.33, and more IAMs than other task-unrelated involuntary thoughts, *t*(220) = 5.06, *p* <.001, *M*_*diff*_ = 1.96, *SE* = 0.35. To assess the theoretical validity, we examined the relationship between self-reported frequency of involuntary autobiographical thoughts about the past and future (as measured by the IAMI questionnaire) and the frequency of involuntary thoughts recorded in the lab. As presented in Table 3, the total IAMI score was positively associated with the total number of involuntary thoughts experienced during the task, *r*(220) = 0.21, *p* =.002, and specifically with the frequency of IAMs, *r*(219) = 0.15, *p* =.02. Further analysis of the IAMI subscales revealed that the past subscale was significantly positively correlated with both the number of IAMs, *r*(219) = 0.20, *p* =.003, and the total number of involuntary thoughts in the lab, *r(*220) = 0.24, *p* <.001. No other bivariate correlations were significant, all *r*s < 0.09, *p*s > 0.165. A full correlation matrix is presented in Table 3.

## Summary and discussion

The IAMI was originally developed as a single-factor scale intended to measure the frequency of involuntary mental time travel, with items assessing the frequency of both past- and future-oriented spontaneous thoughts. However, given the theoretical importance of distinguishing between past- and future-oriented thoughts, a two-factor structure—distinguishing between past and future—was subsequently proposed and empirically tested. The results indicated that this two-factor model, comprising correlated factors for past and future thoughts, provided a better fit than the original single-factor solution (see Berntsen et al.,^24^, Study 1). Accordingly, in the present study, we developed a Polish version of IAMI and assessed its psychometric properties in a controlled laboratory setting. Consistent with the original findings, the two interrelated factors solution showed a good fit to the data, and both the total scale and the individual subscales demonstrated strong internal consistency. The significant positive correlation between the past and future subscales aligns with previous research suggesting that recalling personal past experiences and imagining personal future events rely on shared cognitive processes^54^.

Beyond replicating the factorial structure of the original instrument, Study 1 provides novel evidence for the convergent validity of the IAMI at the laboratory level. Specifically, individual differences captured by the IAMI were related to the occurrence of involuntary autobiographical memories during a vigilance task designed to elicit spontaneous cognition under controlled conditions. This finding extends previous validation work by demonstrating that IAMI scores are not only internally consistent and structurally sound, but also meaningfully related to objectively sampled instances of involuntary thought (Table 3).

Notably, the overall IAMI score was more strongly associated with the frequency of involuntary autobiographical memories than with involuntary future thoughts observed in the laboratory. At first glance, this result appears to contradict earlier findings demonstrating similar frequencies of involuntary thoughts directed toward the past and the future (i.e.^6,55^). One explanation for this discrepancy could be differences in measurement methods. Specifically, diary studies usually reveal comparable frequencies of IAMs and IFTs. However, retrospective self-report methods often indicate a higher frequency of IAMs compared to IFTs^55^. Similarly, as also demonstrated in laboratory studies, individuals report more IAMs than IFTs (e.g.,^11,51^). In that sense, the differences in past and future IAMI can largely be explained by retrospective biases and phenomenological characteristics that facilitate the recall and reporting of past involuntary memories more than involuntary thoughts about the future.

Taken together, Study 1 confirms the reliability and factorial validity of the Polish adaptation of IAMI while also providing the first experimental validation of the scale using laboratory-based indices of involuntary cognition. By employing a vigilance task to elicit involuntary thoughts—an approach previously shown to be both objective and reliable—we provided experimental support that the IAMI captures individual differences that generalize beyond self-report to observable spontaneous thought processes. This multi-level validation strengthens confidence in the scale’s robustness and supports its use in experimental investigations of involuntary autobiographical thinking. In Study 2, we will further examine the construct validity of the Polish IAMI by assessing its associations with related constructs of spontaneous cognition, including mind-wandering, emotional distress, and interoceptive sensitivity.

## Study 2

Having established the factorial structure of the Polish IAMI, we aimed to test the psychometric properties of IAMI in a larger community sample and test its convergent validity using measures of other forms of spontaneous cognition (e.g., mind-wandering), emotional distress and interoception.

Mind-wandering and involuntary past and future thoughts (IAMs and IFTs, respectively) share key similarities in that both occur without conscious intent, relying on associative memory retrieval and self-referential processing^28,55^. However, they differ in key ways: mind-wandering is typically more diffuse and unconstrained, encompassing a broad range of thoughts unrelated to the present task, whereas IAMs and IFTs are more content-specific, often triggered by external cues that evoke past experiences or future simulations^57^. Additionally, it is important to note that mind-wandering can be both deliberate and spontaneous, whereas IAMs and IFTs are by definition spontaneous^58^. Despite these differences, both processes reflect variations of internally directed thought, suggesting a continuum of spontaneous cognition. For that reason, mind-wandering was one of the constructs we used to test the convergent validity of IAMI, expecting a positive relationship, especially with spontaneous mind-wandering.

We also examined depression and anxiety in line with the evidence showing their positive relationship with the frequency of involuntary past and future thoughts (^41,56^). Depression is characterized by negative affectivity and a repetitive focus on distressing thoughts, which has especially been associated with increased frequency of negative involuntary memories and future thoughts^45^. Individuals high in depressive symptoms may experience more frequent intrusive thoughts as negative because mood enhances the accessibility of mood-congruent information^59^. It is also likely that depressed individuals experience rumination, by which they involuntarily recall negative past events and anticipate adverse future outcomes, reinforcing negative affect and cognitive biases that sustain depressive states^60,61^. In this context, research suggests that engaging in co-ruminative discussions with close friends may increase vulnerability to depression by activating maladaptive schemas, such as those related to subjugation, self-sacrifice, approval seeking, pessimism, emotional inhibition, hypercriticalness, and punitiveness. Activation of these schemas can intensify negative mood states, promote behaviors, and trigger involuntary memories and future thoughts that perpetuate or amplify depressive symptoms. Thus rumination in interpersonal settings can contribute to the persistence of negative affect and may influence the frequency of IAMs and IFTs and broader affective processes (e.g., emotion regulation etc.)^62^. Anxiety, on the other hand, is associated with increased vigilance, difficulty in detaching from threat-related information, and intrusive thoughts. Through this, involuntary thoughts may occur more frequently and distressingly, such as in the form of intrusive unpleasant memories or worries about the future^63^. These involuntary cognitions frequently involve anticipations of negative outcomes or recollections of past distressing events, thereby intensifying anxiety symptoms and perpetuating worry and avoidance behaviours^64,65^. Additionally, individuals with anxiety disorders show greater susceptibility to intrusive and involuntary thoughts due to heightened attentional biases toward threat-related stimuli, resulting in persistent and uncontrollable cognitive intrusions that reinforce anxiety symptoms^66^. In line with this, previous research has provided supporting evidence that spontaneous thoughts in anxiety are often intrusive, emotionally charged, and related to perceived threats, such as worry-based mental simulations^6^.

Additionally, we measured interoception, which is the ability to perceive and interpret bodily sensations, and tested its relationship with involuntary thoughts. Previous research has demonstrated the shared cognitive and affective mechanisms between interoceptive awareness and spontaneous cognition^67,68^. Individuals with greater interoceptive awareness tend to experience more vivid or emotionally charged spontaneous thoughts, as bodily sensations can serve as implicit cues for memory retrieval and future simulations. On the other hand, lower interoceptive sensitivity may be associated with dysregulated or distressing spontaneous cognition, such as intrusive thoughts^69,70^. In the present study, the direction of association with the IAMI is expected to be positive: higher interoceptive awareness should relate to a greater frequency of vivid and emotionally salient involuntary past and future thoughts. In line with this, we included interoceptive awareness alongside measures of mind-wandering and emotional distress to support the convergent validity of the IAMI.

### Method

The data was collected as part of EU COST Action CA18106 (The Neural Architecture of Consciousness), which was a large-scale project encompassing magnetic resonance imaging (MRI), electroencephalography and behavioural data collected from healthy neurotypical participants. The study was approved by the local research ethics committee at the Institute of Psychology, Jagiellonian University, Poland (decision no: KE/03/042020).

### Participants

A total of 311 healthy Polish participants took part in the study, aged 19 to 50 years old (111 males, 197 females, 3 other; *M*age = 24.20 years, *SD*age = 4.80). Participants were recruited through an online advertisement and received monetary compensation for their participation in the study. The inclusion criteria required participants to be between 18 and 50 years old and have normal or corrected-to-normal vision, normal hearing, and no history of brain damage or brain surgery.

Since the current sample was recruited as part of a larger study, all participants underwent an MRI scan. Consequently, standard MRI exclusion criteria were applied, including the use of neuropharmacological or other medications affecting neural functioning, the presence of skin diseases, and pregnancy. Additionally, participants with cardiovascular conditions or chronic pain were excluded.

### Measures

#### The Polish adaptation of the IAMI

We used the same Polish version of IAMI that was used in Study 1, which demonstrated it to be a reliable and valid measure of the tendency to experience involuntary past and future thoughts.

#### Mind-wandering: deliberate (MW-D) and Mind-wandering: spontaneous (MW-S) scales

We used the MW-D and MW-S scales^71^ (Polish adaptation by Barzykowski, Zięba, Öner et al.^72^) to assess the frequency of deliberate and spontaneous mind-wandering. Each scale consists of four items designed to capture intentional and unintentional shifts in attention, respectively. The MW-D scale measures the extent to which individuals intentionally disengage from their current task to let their thoughts wander, including items such as “I allow my thoughts to wander on purpose.” In contrast, the MW-S scale assesses the degree to which thoughts drift away from the present activity without conscious control, with statements like “I find my thoughts wandering spontaneously.” Participants rated each item on a seven-point Likert scale (1 = Almost never, 7 = Almost always), with higher scores indicating greater frequency of each type of mind-wandering. These two dimensions reflect distinct mechanisms of attentional control, with spontaneous mind-wandering often linked to reduced executive function and deliberate mind-wandering associated with adaptive reflective thinking.

#### CES. Center for epidemiologic studies depression scale (CES-D)

CES_D^73^ (Polish adaptation by Jankowski^74^ was used to assess depressive symptomatology over the past week. CES-D consists of 20 items, measuring affective, cognitive, and somatic symptoms associated with depression. Participants rated each item on a four-point Likert scale (0 = Rarely or none of the time, 3 = Most or all of the time), with higher scores indicating greater depressive symptoms. Items reflect core aspects of depression, including negative affect, positive affect, somatic complaints, and interpersonal difficulties.

#### Generalized anxiety disorder-7 (GAD-7)

GAD-7^75^ (Polish adaptation by Basińska & Kwissa-Gajewska^76^ was used to assess symptoms of anxiety over the past two weeks. This self-report measure consists of seven items that capture core features of excessive worry, nervousness, and physiological symptoms of anxiety. Participants rated each item on a four-point Likert scale (0 = Not at all to 3 = Nearly every day).

#### The multidimensional assessment of interoceptive awareness (MAIA-2)

MAIA-2 was developed by Mehling et al.^77^ (Polish adaptation by Rogowska et al.^78^) and has been used to assess interoceptive sensibility, a self-report, dispositional measure of tendencies to be internally directed toward the body^79^. The MAIA-2 consists of 37 items across eight subscales: Noticing (awareness of bodily sensations); Not-Distracting (tendency to not ignore uncomfortable sensations); Not-Worrying (reduced anxiety about bodily signals); Attention Regulation (ability to focus on bodily signals); Emotional Awareness (recognizing the link between bodily sensations and emotions); Self-Regulation (using body awareness to regulate distress); Body Listening (attentively focusing on bodily cues); and Trusting (confidence in bodily sensations as reliable). Participants rated each item on a six-point Likert scale (0 = Never to 5 = Always), with higher scores reflecting greater interoceptive awareness. MAIA-2 has been validated as a robust and multidimensional measure of interoceptive sensibility and has also been used in previous studies on mental time travel (e.g.,^65^).

### Procedure

Questionnaires were filled out online. Participants were instructed to complete the questionnaires in a distraction-free environment and were allowed to take breaks as needed. This session also included other questionnaires for different COST Action CA18106 sub-studies that are not part of the present study. The order of questionnaires was semi-random. The total duration of the online session was approximately 90 min.

## Results

### Data screening

As in Study 1, we first screened the data for outliers and conducted normality checks for the study variables. Descriptive indices and Shapiro–Wilk tests for Study 2 were as follows: Future IAMI (*skew* = − 0.79, *SE* = 0.14; *kurtosis* = 1.83, *SE* = 0.28; *W* = 0.95, *p* <.001), Past IAMI (*skew* = − 0.82, *SE* = 0.14; *kurtosis* = 1.78, *SE* = 0.28; *W* = 0.95, *p* <.001), IAMI Total Mean (*skew* = − 0.30, SE = 0.14; *kurtosis* = 0.11, *SE* = 0.28; *W* = 0.98, *p* =.001); MAIA-2 (*skew* = 0.12, SE = 0.14; *kurtosis* = 0.40, SE = 0.28; *W* = 1.00, *p* =.627), Mind-Wandering Deliberate (*skew* = − 0.21, *SE* = 0.14; *kurtosis* = − 0.05, *SE* = 0.28; *W* = 0.99, *p* =.035), Mind-Wandering Spontaneous (*skew* = − 0.36, SE = 0.14; *kurtosis* = − 0.56, *SE* = 0.28; *W* = 0.97, *p* <.001); CES-D (*skew* = 0.60, *SE* = 0.14; *kurtosis* = − 0.19, *SE* = 0.28; *W* = 0.96, *p* <.001); CFQ (*skew* = 0.28, *SE* = 0.14; *kurtosis* = 0.32, *SE* = 0.28; *W* = 0.99, *p* =.060); GAD (*skew* = 0.15, *SE* = 0.14; *kurtosis* = 1.19, *SE* = 0.28; *W* = 0.96, *p* <.001). Thus, MAIA-2 and CFQ showed no evidence of non-normality, whereas the remaining variables exhibited departures from normality. However, consistent with Study 1, all observations were retained because bivariate correlations were robust to moderate deviations from normality.

### Descriptive statistics, internal consistency reliability and factor structure

CFA showed that, as for the original scale, the model with two related factors of past and future subscales yielded a good fit to the data, χ²(158, *N* = 311) = 345, *p* <.001; CFI = 0.94; TLI = 0.92; RMSEA = 0.06, SRMR = 0.05. Internal consistency was also high for both the total IAMI and the past and future subscales, with Cronbach alphas of 0.92, 0.90, 0.87, respectively. A paired-samples t-test indicated that individuals reported more frequent past thoughts than future thoughts, *t*(299) = −8.15, *M*_diff_ = 0.24, *SE* = 0.03, *p* <.001, Cohen’s *d* = 0.47.

### Relationship of IAMI with measures of spontaneous Thoughts, emotional distress and interoception

IAMI total score and scores on past and future subscales were significantly correlated with the mind-wandering scale. All scores were correlated with deliberate and spontaneous mind-wandering, with coefficients ranging from 0.18 to 0.33, *p*s < 0.001 (See Table 4). When we specifically compared the link between IAMI and spontaneous and deliberate mind-wandering, we found that while the correlation of IAMI with spontaneous mind-wandering (*r* =.32, *p* <.001, CI = 0.22-0.042.22.042) was numerically stronger than the correlation with deliberate mind-wandering (*r* =.24, *p* <.001, CI = 0.13-0.0.13.0.34), the difference was not statistically significant (*Z* = 1.15, *p* =.251), indicating that while spontaneous mind-wandering tends to be more associated with IAMI, the effect is not strong enough to be considered meaningful.

**Table 4:** Pearson’s r correlations of IAMI with mind-wandering and emotional distress.

	IAMI Total Mean		IAMI Future		IAMI Past		Mind- Wandering Deliberate		Mind- Wandering Spontaneous		CES-D		GAD Total	
IAMI Total Mean	—													
IAMI Future	0.94	***	—											
IAMI Past	0.94	***	0.82	***	—									
Mind-Wandering_Deliberate	0.24	***	0.21	***	0.22	***	—							
Mind-Wandering_Spontaneous	0.32	***	0.33	***	0.27	***	0.28	***	—					
CES-D	0.17	**	0.16	**	0.19	***	0.37	***	− 0.01		—			
GAD Total	0.48	***	0.42	***	0.48	***	0.28	***	− 0.02		0.57	***	—	
Note. * *p* <.05, ** *p* <.01, *** *p* <.001														

When we examined the relationship between IAMI and emotional distress measures of depression and anxiety, we found strong correlations of total IAMI score with measures of depression, *r(*302) = 0.16, *p* =.004, and anxiety, *r(*309) = 0.48, *p* <.001, showing that, for both the past and future, individuals with higher degrees of depression and anxiety also report more frequent involuntary thoughts.

Lastly, we examined the relationship between IAMI and the interoceptive sensitivity measure of MAIA. We found that IAMI was linked positively with the total score of MAIA, *r(*302) = 0.17, *p* =.002. While IAMI was not significantly correlated with MAIA dimensions of Attention Regulation, Trusting and Not-Distracting, *r* <.08, *p*s > 0.05, for other subscales, the correlations of both total IAMI and past and future subscales ranged from 0.17 to 0.31, *p*s < 0.011. Correlation coefficients are presented in Table 5.

**Table 5:**
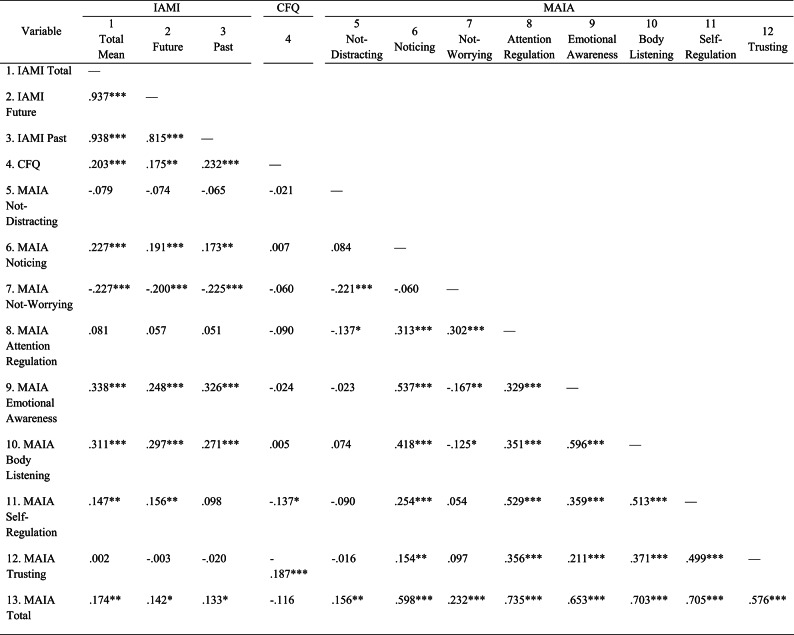
Pearson r correlations among IAMI scores, CFQ, and MAIA-2 subscales.

Note. IAMI = Involuntary Autobiographical Memory Inventory; CFQ = Cognitive Failures Questionnaire; MAIA = Multidimensional Assessment of Interoceptive Awareness. * *p* <.05, ** *p* <.01, *** *p* <.001.

### Summary

To test the convergent validity of the Polish version of IAMI, we examined the relationships between IAMI and mind-wandering, different types of emotional distress and interoception. Our findings indicated a clear relationship between IAMI scores and mind-wandering, highlighting that these constructs, although related, are theoretically distinct. In addition, supporting previous evidence^24,43^, we showed that individuals with higher depression and anxiety reported more frequent experiences of involuntary past and future thoughts on IAMI. We also found that the Polish IAMI was positively associated with interoceptive sensitivity, defined as the total MAIA score, but the few isolated dimensions of interoception (i.e., Attention Regulation, Trusting and Not-Distracting) were not significantly linked. These findings align with recent evidence indicating that individual differences in interoceptive sensibility reliably predict variations in autobiographical memory processes, including involuntary mental time travel^68^. Taken together, Studies 1 and 2 provide complementary evidence for the validity of the IAMI: Study 1 demonstrates that questionnaire scores predict the actual occurrence of involuntary thoughts under controlled laboratory conditions, while Study 2 shows that these individual differences are systematically embedded within broader cognitive–affective and interoceptive profiles.

### General discussion

In the present study, we aimed to develop and examine the convergent validity of a Polish version of the Involuntary Autobiographical Memory Inventory (IAMI)^24^, which measures the frequency of involuntary thoughts about the past and future. The scale has demonstrated significant associations with theoretically related constructs of spontaneous cognition, such as mind-wandering, and involuntary thoughts, thus supporting its convergent validity^24^ and indicating that it reliably captures the intended phenomenon of involuntary mental time travel. Moreover, the scale also showed the expected relationships with emotional factors, as higher levels of emotional distress were linked to more frequent involuntary autobiographical memories, further supporting its sensitivity to relevant constructs.

In the two studies, confirmatory fit analyses using data from independent samples supported the two interrelated-factors structure of the Polish IAMI. The scale showed high item loadings on each factor and internal consistency comparable to that of the original version. In addition, both total IAMI and the past and future subscales showed high internal consistency, suggesting that the Polish IAMI has sufficient statistical properties to be used as a reliable and valid self-report questionnaire to measure the extent to which individuals experience involuntary past and future thoughts.

Notably, the present research goes beyond a standard linguistic adaptation of the IAMI by embedding the validation process within a broader cognitive–experimental framework. By combining questionnaire-based measures with laboratory indices of involuntary thought occurrence and dispositional markers of bodily awareness, the present studies provide a multi-level examination of involuntary mental time travel. This approach allows the Polish adaptation of the IAMI to function not only as a measurement tool, but also as a vehicle for testing theoretically relevant predictions about spontaneous past and future thinking.

In structuring the discussion, we first examine convergent validity using laboratory-based data from Study 1, followed by questionnaire-based evidence from Study 2, which extends understanding of the associations between spontaneous past and future thinking and related constructs such as mind-wandering and interoceptive sensitivity. We then conclude by situating these findings within a broader theoretical framework, highlighting their implications for advancing knowledge of autobiographical memory retrieval.

### Convergent validity using laboratory studies

When studying involuntary thoughts, researchers have typically relied on either self-report questionnaires or diary methods, asking participants to reflect on and report how often they experience involuntary thoughts^6,80,81^. While these measures are valuable, they can be influenced by retrospective biases or differences in how well individuals remember their own spontaneous thoughts. For that reason, in the present study we employed a well-established laboratory-based procedure to measure involuntary thoughts (e.g.,^11,12,32–34^), which also allowed us to validate the self-report IAMI.

As we expected, IAMI was significantly associated with the frequency of involuntary thoughts observed in the laboratory settings. Although the future subscale of IAMI was not linked with the number of future thoughts, there was a significant link with the total number of involuntary thoughts. One reason for this might be the salience of past representations compared to future thoughts. Remembering draws on concrete perceptual details, whereas future thinking involves piecing together memories to imagine possible events^82^. As a result, people’s judgments about their future thinking may not accurately reflect their actual experiences. In that sense, the future dimension of IAMI may be less sensitive than the past dimension; however, its significant relationship with the overall number of involuntary thoughts recorded in the laboratory setting supports its validity for real-time assessment of spontaneous thoughts.

Overall, while IAMI is valuable as a self-report scale that captures how frequently people experience involuntary past and future thoughts, connecting these subjective ratings to real-time observations further strengthens its credibility. This convergence between subjective and objective methods indicates IAMI’s validity, enhancing its overall usefulness in spontaneous cognition research.

### Convergent validity using mind-wandering and emotional distress measures

The pattern of associations obtained in the current study offers further support for the theoretical distinctiveness of involuntary thoughts as measured by IAMI. As expected, we observed strong positive correlations between IAMI scores and mind-wandering tendencies, which supports previous evidence showing the conceptual and empirical relationship between spontaneous thought generation and involuntary mental time travel^57,83^. Considering their conceptual relationship, we expected involuntary thoughts to be more closely related to spontaneous mind-wandering than to deliberate mind-wandering. In line with this, while the strength of the relationship was different, this difference did not reach statistical significance.

This comparable strength of the association between involuntary thoughts and both spontaneous and deliberate mind-wandering could be explained by considering the shared cognitive mechanisms underlying different forms of internally generated thought. Specifically, spontaneous involuntary memories and both types of mind-wandering may rely on common cognitive processes, such as attention, associative processing, and transient reductions in cognitive control^66^. These mechanisms (e.g., the lack of cognitive control) may facilitate spontaneous intrusions into consciousness, irrespective of the intentionality involved in initiating such episodes. Moreover, it is worth mentioning that drawing the line between involuntary and intentional mind-wandering is difficult in itself (if possible), and the mind-wandering scale reflects this difficulty. Many items present in the deliberate mind-wandering subscale (e.g., “I enjoy mind-wandering.”, “I find mind-wandering is a good way to cope with boredom.”) could also apply to people who experience *involuntary* mind-wandering but have a positive attitude toward the occurrence of such experiences and/or their contents. Thus, rather than viewing spontaneous and deliberate mind-wandering as entirely separate phenomena, we suggest they might be best conceptualized as existing along a continuum, sharing underlying cognitive resources that promote involuntary memories.

Importantly, the frequency of involuntary memories and future thoughts reported via IAMI was also positively associated with emotional distress, particularly symptoms of depression and anxiety. These findings are consistent with previous evidence suggesting that individuals experiencing elevated levels of distress tend to encounter more frequent and intrusive spontaneous memories^43,84,85^.

Additionally, our findings expand on prior research by demonstrating a significant relationship between IAMI scores and interoceptive sensitivity^68^. Defined as the capacity to perceive and integrate internal bodily signals, interoception is central to self-related bodily experiences such as body ownership and agency (i.e.^86^,). Considering this centrality, heightened interoceptive sensitivity may enable individuals to more readily detect involuntary thoughts as they spontaneously arise in their stream of consciousness. In other words, individuals who are more attuned to subtle bodily sensations might also be more sensitive to detecting spontaneous thoughts. It is also possible that greater interoceptive sensitivity indirectly triggers involuntary thoughts by enhancing the emotional intensity of experiences. More specifically, individuals with greater interoceptive sensitivity tend to have enhanced emotional awareness following emotionally charged experiences, particularly negative ones^87,88^. Greater emotional clarity and intensity may heighten the salience of negative events, making them more vivid and accessible in memory, and increasing the chance that affective cues will trigger involuntary thoughts^89,90^. Here, although not all dimensions of interoception showed robust associations, particularly for those more related to confidence regarding interoceptive experiences, we observed that individuals with greater overall sensitivity to internal bodily states were more likely to report frequent involuntary thoughts. These findings not only support the validity of the IAMI but also extend previous evidence by demonstrating a significant relationship between IAMI scores and interoceptive sensitivity — suggesting that bodily awareness may serve as a gateway (i.e., trigger), facilitating the recognition and reporting of involuntary autobiographical memories and future-oriented thoughts.

### Theoretical implications

The relationship observed in the present study—as well as in the recent study by Messina and Berntsen^68^—between interoceptive sensitivity and involuntary retrieval provides further support for the threshold model proposed and discussed in detail by Barzykowski and colleagues^27,91–95^. The threshold model addresses the question of how autobiographical memories are made aware. Its core assumption is that for a memory to become recalled, it must surpass a certain awareness threshold. The ease with which this occurs—referred to as its accessibility—can be influenced by various characteristics of the memory itself, such as its emotional intensity, personal significance, or the physiological reactions it evokes. According to the model, “phenomenologically rich” memories—those that are vivid, emotional, intense, causing physiological reactions—are more likely to exceed this threshold because they are more attention-grabbing and more likely to disrupt ongoing cognitive processes. The phrase “phenomenologically rich” is used metaphorically to describe the qualities that make certain memories more accessible and more prone to involuntary retrieval, even in the absence of intentional search. A key assumption of the model is that the awareness threshold is not fixed but can be modulated by various factors, including metacognitive factors such as retrieval intentionality (i.e., the intention and/or effort to recall a memory). The present findings, along with those of Messina and Berntsen^68^, suggest that individual differences in interoceptive sensitivity—awareness of internal bodily signals—may also influence this threshold. Specifically, individuals with heightened interoceptive sensitivity may have a lower awareness threshold, making them more likely to detect and attend to spontaneously occurring mental contents. Additionally, they may possess a larger pool of highly accessible cues (e.g., highly emotional ones) that are particularly effective in triggering involuntary memories. This suggests that bodily cues may serve as amplifiers in the retrieval of involuntary autobiographical memories. Taken together, these findings not only support the threshold model but also extend its assumptions by introducing an additional mechanism: the role of individual interoceptive sensitivity. This highlights a new direction in understanding the processes underlying autobiographical memory retrieval, suggesting that bodily awareness may be another factor that shapes the awareness of memories—an idea that resonates well with the broader concept of embodied cognition.

### Limitations

Our studies have some limitations that do not significantly undermine the present results but may be considered in future research. In particular, the sample size in Study 1 (*N* = 222) was relatively modest for confirmatory factor analysis, and although Study 2 involved a larger sample (*N* = 311), this number may still limit the scope of examining convergent validity with related constructs. Nevertheless, even with relatively large, though not enormous, samples, we demonstrated satisfactory psychometric properties of the IAMI. This provides clear evidence that the Polish adaptation of the IAMI is a reliable and valid tool for assessing individual differences in involuntary mental time travel and can be confidently applied in future studies. Larger and more diverse samples would be valuable to further strengthen the robustness and generalizability of the Polish IAMI.

### Conclusions and future directions

The present study makes several novel contributions to the literature on involuntary autobiographical memory and future thought. First, it is the first adaptation of the Involuntary Autobiographical Memory Inventory (IAMI) into Polish, addressing an important gap in cross-cultural research on spontaneous thought processes. Importantly, our validation went beyond standard procedures by combining subjective self-report data with objective laboratory measures, using a computerized vigilance task adapted from validated paradigms (e.g.,^31–35^). This integration of methods provides a more comprehensive and robust validation framework than prior adaptations, advancing the field by bridging laboratory-based and self-report approaches. Second, confirmatory factor analyses in two independent samples consistently supported the two-factor structure (past and future involuntary thoughts), replicating the original factor solution and demonstrating the robustness of the Polish version. The scale further showed strong item loadings, high internal consistency, and reliability comparable to the original measure, thereby confirming its psychometric soundness. Third, our findings provide new evidence of significant associations between IAMI scores and interoceptive sensitivity, highlighting a previously underexplored link between bodily awareness and the occurrence of involuntary autobiographical memories and future-oriented thoughts. Taken together, these strengths underscore that the Polish adaptation of the IAMI is not only a reliable and valid instrument but also extends the literature by providing unique insights into the embodied nature of spontaneous cognition in a new cultural context.

More broadly, the findings corroborate not only the convergent validity of the Polish IAMI, but also previous evidence regarding the cognitive and emotional context in which involuntary mental time travel occurs. Moreover, they highlight a novel direction for understanding the processes underlying autobiographical memory retrieval, suggesting that bodily awareness may be an additional factor shaping the emergence of memories into consciousness—an idea that aligns with the broader concept of embodied cognition. Future research should aim to further investigate these complex mechanisms. We believe that future research integrating self-report measures such as IAMI with moment-to-moment experience sampling during task performance may help bridge the gap between dispositional tendencies and real-time cognitive dynamics. For example, individuals with high IAMI scores may show a higher frequency of or richer phenomenological detail in involuntary thoughts captured during a vigilance task. These designs could provide more process-level insights into how involuntary thoughts are shaped by factors like cognitive load, the emotional salience of cues, or individual differences in bodily awareness. Finally, the present findings underscore the value of multi-level approaches that integrate self-report, experimental, and bodily-level indices of involuntary cognition. Such approaches may be particularly informative for future research on autobiographical memory, mental time travel, and spontaneous thought, including clinical and developmental investigations. By demonstrating that involuntary past and future thoughts can be meaningfully assessed across converging levels of analysis, the present work contributes to a more comprehensive understanding of the mechanisms underlying spontaneous (autobiographical) cognition and related phenomena, including déjà vu, cognitive style, and mindfulness (e.g.,^96–99^).

## Supplementary Information

Below is the link to the electronic supplementary material.

Supplementary Material 1

## Data Availability

The data that support the findings of this study will be made available upon a request. For any additional information or specific inquiries regarding the data, please contact the corresponding Author.

## References

[1] Tulving, E. Episodic memory: from Mind to brain. *Annu. Rev. Psychol.* **53**, 1–25 (2002). 11752477 10.1146/annurev.psych.53.100901.135114

[2] Berntsen, D. Voluntary and involuntary access to autobiographical memory. *Memory* **6**, 113–141 (1998). 9640425 10.1080/741942071

[3] Berntsen, D. & Jacobsen, A. S. Involuntary (spontaneous) mental time travel into the past and future. *Conscious. Cogn.* **17**, 1093–1104 (2008). 18424178 10.1016/j.concog.2008.03.001

[4] Schacter, D. L. & Addis, D. R. On the constructive episodic simulation of past and future events. *Behav. Brain Sci.* **30**, 331–332 (2007).

[5] Berntsen, D. *Involuntary Autobiographical Memories: an Introduction To the Unbidden Past* (Cambridge Univ. Press, 2009).

[6] Finnbogadóttir, H. & Berntsen, D. Involuntary future projections are as frequent as involuntary memories, but more positive. *Conscious. Cogn.* **22**, 272–280 (2013). 22884775 10.1016/j.concog.2012.06.014

[7] Rasmussen, A. S., Ramsgaard, S. B. & Berntsen, D. Frequency and functions of involuntary and voluntary autobiographical memories across the day. *Psychol. Conscious.* **2**, 185–205 (2015).

[8] Schlagman, S., Kvavilashvili, L. & Schultz, J. Effects of age on involuntary autobiographical memories. In Involuntary Memory (ed Mace, J. H.) 1–19 (Blackwell, (2007).

[9] Schlagman, S. et al. Differential effects of age on involuntary and voluntary autobiographical memory. *Psychol. Aging*. **24**, 397–411 (2009). 19485657 10.1037/a0015785

[10] Barzykowski, K. & Niedźwieńska, A. Involuntary autobiographical memories are relatively more often reported during high cognitive load tasks. *Acta Psychol.* **182**, 119–128 (2018). 10.1016/j.actpsy.2017.11.014 29169060

[11] Barzykowski, K., Hajdas, S., Radel, R. & Kvavilashvili, L. Effects of inhibitory control capacity and cognitive load on involuntary past and future thoughts: a laboratory study. *Conscious. Cogn.* **102**, 103353 (2022). 35642842 10.1016/j.concog.2022.103353

[12] Vannucci, M. et al. Why are we not flooded by involuntary autobiographical memories? Few cues are more effective than many. *Psychol. Res.* **79**, 1077–1085 (2015). 25468208 10.1007/s00426-014-0632-y

[13] Mace, J. H. & Atkinson, E. Can we determine the functions of everyday involuntary autobiographical memories? In Applied Memory (ed Kelley, M. R.) 199–212 (Nova Science, (2009).

[14] Mace, J. H. Are involuntary autobiographical memory and deja vu cognitive failures? Commentary on Barzykowski & Moulin (2023a). *Behav. Brain Sci. ***46**, E356 (2023). 10.1017/S0140525X23000213 37961774

[15] Barzykowski, K. & Moulin, C. Further advancing theories of retrieval of the personal past. *Behav. Brain Sci.* **46**, E384 (2023). 37961810 10.1017/S0140525X23002765

[16] Berntsen, D. The unbidden past: involuntary autobiographical memories as a basic mode of remembering. *Curr. Dir. Psychol. Sci.* **19**, 138–142 (2010).

[17] Rasmussen, A. S. & Berntsen, D. The possible functions of involuntary autobiographical memories. *Appl. Cogn. Psychol.* **23**, 1137–1152 (2009).

[18] Pillemer, D. Directive functions of autobiographical memory: the guiding power of the specific episode. *Memory* **11**, 193–202 (2003). 12820831 10.1080/741938208

[19] Walker, W. R., Skowronski, J. J. & Thompson, C. P. Life is pleasant-and memory helps to keep it that way! *Rev. Gen. Psychol.* **7**, 203–210 (2003).

[20] Ehlers, A., Hackmann, A. & Michael, T. Intrusive re-experiencing in post-traumatic stress disorder: phenomenology, theory, and therapy. *Memory* **12**, 403–415 (2004). 15487537 10.1080/09658210444000025

[21] van der Kolk, B. A. & Fisler, R. Dissociation and the fragmentary nature of traumatic memories: overview and exploratory study. *J. Trauma. Stress*. **8**, 505–525 (1995). 8564271 10.1007/BF02102887

[22] Pile, V. & Lau, J. Y. F. Intrusive images of a distressing future: links between prospective mental imagery, generalized anxiety and a tendency to suppress emotional experience in youth. *Behav. Res. Ther.* **124**, 103508 (2020). 31855697 10.1016/j.brat.2019.103508

[23] Verfaellie, M. et al. Future thinking in PTSD: preliminary evidence for altered event construction. *Psychiatry Res.* **333**, 115768 (2024). 38325161 10.1016/j.psychres.2024.115768 PMC10901291

[24] Berntsen, D., Rubin, D. C. & Salgado, S. The frequency of involuntary autobiographical memories and future thoughts in relation to daydreaming, emotional distress, and age. *Conscious. Cogn.* **36**, 352–372 (2015). 26241025 10.1016/j.concog.2015.07.007 PMC4552601

[25] Shan, Y., Rubin, D. C. & Berntsen, D. Involuntary autobiographical memories as a transdiagnostic factor in mental disorders. *Clin. Psychol. Rev.* **116**, 102545 (2025). 39874680 10.1016/j.cpr.2025.102545

[26] Berntsen, D. & Hall, N. M. The episodic nature of involuntary autobiographical memories. *Mem. Cogn.* **32**, 789–803 (2004). 10.3758/bf03195869 15552356

[27] Barzykowski, K. & Staugaard, S. R. Does retrieval intentionality really matter? Similarities and differences between involuntary memories and directly and generatively retrieved voluntary memories. *Br. J. Psychol.* **107**, 519–536 (2016). 26514399 10.1111/bjop.12160

[28] Kvavilashvili, L. & Rummel, J. On the nature of everyday prospection: a review and theoretical integration of research on mind-wandering, future thinking, and prospective memory. *Rev. Gen. Psychol.* **24**, 210–237 (2020).

[29] Berntsen, D., Staugaard, S. R. & S√łrensen, L. M. T. Why am I remembering this now? Predicting the occurrence of involuntary (spontaneous) episodic memories. *J. Exp. Psychol. Gen.* **142**, 426–444 (2013). 22746701 10.1037/a0029128

[30] Ball, C. T. Can we elicit involuntary autobiographical memories in the laboratory? In Involuntary Memory (ed Mace, J. H.) 127–152 (Blackwell, (2007).

[31] Barzykowski, K. et al. The role of inhibitory control and ADHD symptoms in the occurrence of involuntary thoughts about the past and future: an individual differences study. *Conscious. Cogn.* **95**, 103208 (2021). 34601354 10.1016/j.concog.2021.103208

[32] Schlagman, S. & Kvavilashvili, L. Involuntary autobiographical memories in and outside the laboratory: how different are they from voluntary autobiographical memories? Mem. *Cogn* **36**, 920–932 (2008). 10.3758/mc.36.5.920 18630199

[33] Vannucci, M. et al. Visual attentional load affects the frequency of involuntary autobiographical memories and their level of meta-awareness. *Mem. Cogn.* **47**, 117–129 (2019). 10.3758/s13421-018-0854-0 30191407

[34] Mazzoni, G. Involuntary memories and involuntary future thinking differently tax cognitive resources. *Psychol. Res.* **83**, 684–697 (2019). 30478607 10.1007/s00426-018-1123-3

[35] Mazzoni, G., Vannucci, M. & Batool, I. Manipulating cues in involuntary autobiographical memory: verbal cues are more effective than pictorial cues. *Mem. Cogn.* **42**, 1076–1085 (2014). 10.3758/s13421-014-0420-3 24871426

[36] Kamiya, S. Relationship between frequency of involuntary autobiographical memories and cognitive failure. *Memory* **22**, 839–851 (2014). 24161129 10.1080/09658211.2013.838630

[37] Kvavilashvili, L. & Mandler, G. Out of one’s mind: a study of involuntary semantic memories. *Cogn. Psychol.* **48**, 47–94 (2004). 14654036 10.1016/s0010-0285(03)00115-4

[38] Mace, J. H. Priming involuntary autobiographical memories. *Memory* **13**, 874–884 (2005). 16298894 10.1080/09658210444000485

[39] Mace, J. H. et al. Accuracy and perspective in involuntary autobiographical memory. *Appl. Cogn. Psychol.* **25**, 20–28 (2011).

[40] Ibaceta, M. & Madrid, H. P. Personality and mind-wandering self-perception: the role of meta-awareness. *Front. Psychol.* **12**, 581129 (2021). 33935848 10.3389/fpsyg.2021.581129 PMC8081844

[41] Robison, M. K., Gath, K. I. & Unsworth, N. The neurotic wandering mind: an individual differences investigation of neuroticism, mind-wandering, and executive control. *Q. J. Exp. Psychol.* **70**, 649–663 (2017). 10.1080/17470218.2016.1145706 26821933

[42] Klein, K. & Boals, A. The relationship of life event stress and working memory capacity. *Appl. Cogn. Psychol.* **15**, 565–579 (2001).

[43] Brewin, C. R. et al. Intrusive images in psychological disorders: characteristics, neural mechanisms, and treatment implications. *Psychol. Rev.* **117**, 210–232 (2010). 20063969 10.1037/a0018113 PMC2834572

[44] Li, H. X. et al. Characterizing human spontaneous thoughts and its application in major depressive disorder. *J. Affect. Disord*. **365**, 276–284 (2024). 39147154 10.1016/j.jad.2024.08.060

[45] Kvavilashvili, L. & Schlagman, S. Involuntary autobiographical memories in dysphoric mood: a laboratory study. *Memory* **19**, 331–345 (2011). 21678152 10.1080/09658211.2011.568495

[46] Merino, H., Senra, C. & Ferreiro, F. Are worry and rumination specific pathways linking neuroticism and symptoms of anxiety and depression in patients with generalized anxiety disorder, major depressive disorder and mixed anxiety-depressive disorder? *PLoS One*. **11**, e0156169 (2016). 27243462 10.1371/journal.pone.0156169 PMC4886972

[47] Perkins, A. M. et al. Thinking too much: self-generated thought as the engine of neuroticism. *Trends Cogn. Sci.* **19**, 492–498 (2015). 26320724 10.1016/j.tics.2015.07.003

[48] Del Palacio-Gonzalez, A. & Berntsen, D. The tendency for experiencing involuntary future and past mental time travel is robustly related to thought suppression: an exploratory study. *Psychol. Res.* **83**, 788–804 (2019). 30569386 10.1007/s00426-018-1132-2

[49] Mace, J. H. (ed) *Involuntary Memory* (Blackwell, 2007).

[50] Vigliocco, G. et al. Language and imagery: effects of Language modality. *Proc. Biol. Sci.* **272**, 1859–1863 (2005). 16096100 10.1098/rspb.2005.3169 PMC1559869

[51] Plimpton, B., Patel, P. & Kvavilashvili, L. Role of triggers and dysphoria in mind-wandering about past, present and future: a laboratory study. *Conscious. Cogn.* **33**, 261–276 (2015). 25676320 10.1016/j.concog.2015.01.014

[52] Barzykowski, K. et al. The role of working memory in the occurrence of involuntary thoughts about the past and future: an experimental investigation. *J. Exp. Psychol. Learn. Mem. Cogn.* (in press). 10.1037/xlm0001624 42574137

[53] The jamovi project. jamovi (Version 2.3). (2021). https://www.jamovi.org

[54] Szpunar, K. K. Episodic future thought: an emerging concept. *Perspect. Psychol. Sci.* **5**, 142–162 (2010). 26162121 10.1177/1745691610362350

[55] Branch, J. G. Individual differences in the frequency of voluntary & involuntary episodic memories, future thoughts, and counterfactual thoughts. *Psychol. Res.* **87**, 2171–2182 (2023). 36781455 10.1007/s00426-023-01802-2

[56] Yeung, R. C. & Fernandes, M. A. Recurrent involuntary memories and Mind wandering are related but distinct. *Psychol. Res.* **88**, 1483–1498 (2024). 38652302 10.1007/s00426-024-01961-w

[57] Berntsen, D. Involuntary autobiographical memories and their relation to other forms of spontaneous thoughts. *Philos. Trans. R Soc. Lond. B Biol. Sci.* **376**, 20190693 (2021). 33308074 10.1098/rstb.2019.0693 PMC7741080

[58] Seli, P., Carriere, J. S. & Smilek, D. Not all Mind wandering is created equal: dissociating deliberate from spontaneous Mind wandering. *Psychol. Res.* **79**, 750–758 (2015). 25284016 10.1007/s00426-014-0617-x

[59] Faul, L., Baumann, M. G. & LaBar, K. S. The representation of emotional experience from imagined scenarios. *Emotion* **23**, 1670–1686 (2023). 36395023 10.1037/emo0001192 PMC10188652

[60] Nolen-Hoeksema, S., Wisco, B. E. & Lyubomirsky, S. Rethinking rumination. *Perspect. Psychol. Sci.* **3**, 400–424 (2008). 26158958 10.1111/j.1745-6924.2008.00088.x

[61] Marchetti, I., Koster, E. H., Sonuga-Barke, E. J. & De Raedt, R. The default mode network and recurrent depression: a Neurobiological model of cognitive risk factors. *Neuropsychol. Rev.* **22**, 229–251 (2012). 22569771 10.1007/s11065-012-9199-9

[62] Balsamo, M. et al. The mediating role of early maladaptive schemas in the relation between co-rumination and depression in young adults. *PLoS One*. **10**, e0140177 (2015). 26488748 10.1371/journal.pone.0140177 PMC4619064

[63] Hallford, D., Seydavi, M. & Akbari, M. The perceived functions and phenomenological characteristics of future thinking and clinically significant generalized anxiety disorder symptoms. *Clin. Psychol. Psychother.* **31**, e2978 (2024). 38706135 10.1002/cpp.2978

[64] Hallford, D. J. et al. Impairments in episodic future thinking for positive events and anticipatory pleasure in major depression. *J. Affect. Disord*. **260**, 536–543 (2020). 31539690 10.1016/j.jad.2019.09.039

[65] Berntsen, D. & Rubin, D. C. Pretraumatic stress reactions in soldiers deployed to Afghanistan. *Clin. Psychol. Sci.* **3**, 663–674 (2015). 26366328 10.1177/2167702614551766 PMC4564108

[66] Hirsch, C. R. & Mathews, A. A cognitive model of pathological worry. *Behav. Res. Ther.* **50**, 636–646 (2012). 22863541 10.1016/j.brat.2012.06.007 PMC3444754

[67] Christoff, K. et al. Mind-wandering as spontaneous thought: a dynamic framework. *Nat. Rev. Neurosci.* **17**, 718–731 (2016). 27654862 10.1038/nrn.2016.113

[68] Messina, A. & Berntsen, D. Self-reported sensibility to bodily signals predicts individual differences in autobiographical memory: an exploratory study. *Memory* **32**, 996–1011 (2024). 38990765 10.1080/09658211.2024.2373891

[69] Kalivas, P. W., Gourley, S. L. & Paulus, M. P. Intrusive thinking: circuit and synaptic mechanisms of a transdiagnostic psychiatric symptom. *Neurosci. Biobehav Rev.* **150**, 105196 (2023). 37094741 10.1016/j.neubiorev.2023.105196 PMC10249786

[70] Koenen, L. R. et al. Associative learning and extinction of conditioned threat predictors across sensory modalities. *Commun. Biol.* **4**, 553 (2021). 33976383 10.1038/s42003-021-02008-1 PMC8113515

[71] Carriere, J. S. A., Seli, P. & Smilek, D. Wandering in both Mind and body: individual differences in Mind wandering and inattention predict fidgeting. *Can. J. Exp. Psychol.* **67**, 19–31 (2013). 23458548 10.1037/a0031438

[72] Barzykowski, K., Zięba, S., Öner, S., Ilczuk, E., Jakubowska, W., Hobot, J. & Wierzchoń, M. Measuring the wandering mind: Polish psychometric adaptation of mind-wandering scales and their links to cognitive control. *Acta Psychologica* (under revision). 10.1016/j.actpsy.2026.107503 42485813

[73] Radloff, L. S. The CES-D scale: a self-report depression scale for research in the general population. *Appl. Psychol. Meas.* **1**, 385–401 (1977).

[74] Jankowski, K. S. Morningness-eveningness and depressive symptoms: test on the components level with CES-D in Polish students. *J. Affect. Disord*. **196**, 47–53 (2016). 26897456 10.1016/j.jad.2016.02.015

[75] Spitzer, R. L., Kroenke, K., Williams, J. B. & Löwe, B. A brief measure for assessing generalized anxiety disorder: the GAD-7. *Arch. Intern. Med.* **166**, 1092–1097 (2006). 16717171 10.1001/archinte.166.10.1092

[76] Basińska, B. A. & Kwissa-Gajewska, Z. Psychometric properties of the Polish version of the generalized anxiety disorder scale (GAD-7) in a non-clinical sample of employees during pandemic crisis. *Int. J. Occup. Med. Environ. Health*. **36**, 493–504 (2023). 37737500 10.13075/ijomeh.1896.02104 PMC10691419

[77] Mehling, W. E. et al. The multidimensional assessment of interoceptive awareness, version 2 (MAIA-2). *PLoS One*. **13**, e0208034 (2018). 30513087 10.1371/journal.pone.0208034 PMC6279042

[78] Rogowska, A. M., Tataruch, R. & Klimowska, K. Validation of the shortened 24-item multidimensional assessment of interoceptive awareness, version 2 (Brief MAIA-2). *Sci. Rep.* **13**, 21270 (2023). 38042880 10.1038/s41598-023-48536-0 PMC10693589

[79] Garfinkel, S. N. et al. Knowing your own heart: distinguishing interoceptive accuracy from interoceptive awareness. *Biol. Psychol.* **104**, 65–74 (2015). 25451381 10.1016/j.biopsycho.2014.11.004

[80] Rasmussen, A. S. & Berntsen, D. The unpredictable past: spontaneous autobiographical memories outnumber autobiographical memories retrieved strategically. *Conscious. Cogn.* **20**, 1842–1846 (2011). 21852157 10.1016/j.concog.2011.07.010

[81] Rubin, D. C. & Berntsen, D. The frequency of involuntary and voluntary autobiographical memories across the life span. *Mem. Cogn.* **37**, 679–688 (2009). 10.3758/37.5.679 PMC3044938 19487759

[82] Addis, D. R., Wong, A. T. & Schacter, D. L. Age-related changes in the episodic simulation of future events. *Psychol. Sci.* **19**, 33–41 (2008). 18181789 10.1111/j.1467-9280.2008.02043.x

[83] Krans, J., de Bree, J. & Moulds, M. L. Involuntary cognitions in everyday life: exploration of type, quality, content, and function. *Front. Psychiatry*. **6**, 7 (2015). 25698979 10.3389/fpsyt.2015.00007 PMC4313579

[84] Marks, E. H., Franklin, A. R. & Zoellner, L. A. Can’t get it out of my mind: a systematic review of predictors of intrusive memories of distressing events. *Psychol. Bull.* **144**, 584 (2018). 29553763 10.1037/bul0000132 PMC5938103

[85] Gehrt, T. B. et al. Autobiographical memory and episodic future thinking in severe health anxiety: a comparison with obsessive-compulsive disorder. *Cogn. Ther. Res.* **44**, 89–107 (2020).

[86] Blanke, O., Slater, M. & Serino, A. Behavioral, neural, and computational principles of bodily self-consciousness. *Neuron* **88**, 145–166 (2015). 26447578 10.1016/j.neuron.2015.09.029

[87] Craig, A. D. How do you feel-now? The anterior Insula and human awareness. *Nat. Rev. Neurosci.* **10**, 59–70 (2009). 19096369 10.1038/nrn2555

[88] Edwards, D. J. & Lowe, R. Associations between mental health, interoception, psychological flexibility, and self-as-context, as predictors for alexithymia: a deep artificial neural network approach. *Front. Psychol.* **12**, 637802 (2021). 33868110 10.3389/fpsyg.2021.637802 PMC8044902

[89] Ianì, F. Embodied memories: reviewing the role of the body in memory processes. *Psychon Bull. Rev.* **26**, 1747–1766 (2019). 31654375 10.3758/s13423-019-01674-x

[90] Matsumoto, N. et al. Subjective judgments on direct and generative retrieval of autobiographical memory: the role of interoceptive sensibility and emotion. *Mem. Cogn.* **51**, 1–20 (2022). 10.3758/s13421-022-01280-8 35294741

[91] Barzykowski, K. & Staugaard, S. R. How intention and monitoring your thoughts influence characteristics of autobiographical memories. *Br. J. Psychol.* **109**, 321–340 (2018). 28872657 10.1111/bjop.12259

[92] Barzykowski, K., Niedźwieńska, A. & Mazzoni, G. How intention to retrieve a memory and expectation that it will happen influence retrieval of autobiographical memories. *Conscious. Cogn.* **72**, 31–48 (2019). 31078046 10.1016/j.concog.2019.03.011

[93] Barzykowski, K. & Mazzoni, G. Do intuitive ideas of the qualities that should characterize involuntary and voluntary memories affect their classification? Psychol. *Res* **86**, 170–195 (2022). 10.1007/s00426-020-01465-3 PMC8821514 33582862

[94] Moulin, C. J. A., Carreras, F. & Barzykowski, K. The phenomenology of autobiographical retrieval. *Wiley Interdiscip Rev. Cogn. Sci.* **14**, e1638 (2023). 36458642 10.1002/wcs.1638

[95] Carreras, F., Staugaard, S. R., Moulin, C. & Barzykowski, K. Unravelling autobiographical memory retrieval: processes and phenomenology. In *The Handbook of Autobiographical Memory: From Basic to Applied Perspectives* (eds. El Haj, M. et al.) (in press).

[96] Barzykowski, K. & Moulin, C. Are involuntary autobiographical memory and déjà Vu natural products of memory retrieval? Behav. *Brain Sci.* **46**, E356 (2023). 10.1017/S0140525X22002035 36111499

[97] Zareen, G., Souchay, C., Barzykowski, K. & Moulin, C. J. A. Spontaneous metacognitive experiences and involuntary memories in the laboratory. *Conscious. Cogn.* **137**, 103976 (2026). 41401545 10.1016/j.concog.2025.103976

[98] Matsumoto, N. et al. Shinshu mindful study: can mindfulness training change the retrieval mode of autobiographical memory? *Memory* **34**, 1–19 (2026). 41664487 10.1080/09658211.2026.2628213

[99] Kvamme, T. L. et al. An inwardly focused cognitive style links mental imagery and0020mental health. *Heliyon* **12**, e44433 (2026).

