# Supplementary material for: Convergent validation of the Involuntary Autobiographical Memory Inventory across levels of analysis in a Polish sample
Source: Sci Rep. 2026 May 19;16:22862. doi: 10.1038/s41598-026-40606-3 (PMC13388914; doi:10.1038/s41598-026-40606-3)
Supplement: Supplementary file 1 — Supplementary Material 1 [file 41598_2026_40606_MOESM1_ESM.pdf]

Inwentarz Wspomnień Mimowolnych (Berntsen et al., 2016)

Poniższe pytania dotyczą tego, jak często wspomnienia i myśli dotyczące przyszłości przychodzą Ci na myśl w sposób spontaniczny (bez próbowania ich przypominania) na co dzień. Dla każdego pytania wybierz odpowiedź, która w najlepszy sposób odpowiada temu jak często doświadczasz tego typu treści

| | Nigdy | Raz na miesiąc lub częściej | Raz na tydzień lub częściej | Raz na dzień lub częściej | Raz na godzinę lub częściej |
|---|---|---|---|---|---|
| 1. Gdy odpoczywam lub wykonuję rutynowe czynności, wyobrażenia dotyczące przyszłych wydarzeń same przychodzą mi na myśl – choć nie próbuję ich sobie przypominać celowo | 0 | 1 | 2 | 3 | 4 |
| 2. Wspomnienia dotyczące mojej osobistej przeszłości przychodzą mi same na myśl – choć nie próbuję ich sobie przypominać | 0 | 1 | 2 | 3 | 4 |
| 3. Niektóre miejsca i lokalizacje wywołują wyobrażenia dotyczące przyszłych wydarzeń – choć nie próbuję ich sobie przypominać celowo | 0 | 1 | 2 | 3 | 4 |
| 4. Gdy dzieje się coś nieoczekiwanego, mimowolnie to pamiętam, choć nie staram się sobie tego przypominać. Po prostu przypomina mi się to | 0 | 1 | 2 | 3 | 4 |
| 5. Wyobrażenia dotyczące przyszłych wydarzeń przychodzą mi same w myślach – choć nie próbuję ich sobie przywoływać celowo | 0 | 1 | 2 | 3 | 4 |
| 6. Niektóre emocje, nastroje albo myśli przywołują wyobrażenia dotyczące przyszłych wydarzeń – choć nie próbuję ich sobie przywoływać celowo | 0 | 1 | 2 | 3 | 4 |
| 7. Gdy się nudzę, wyobrażenia dotyczące przyszłych wydarzeń same przychodzą mi na myśl – choć nie próbuję ich sobie przywoływać celowo | 0 | 1 | 2 | 3 | 4 |
| 8. Po tym, jak doświadczyłam/łem czegoś, co zrobiło na mnie duże wrażenie, mimowolnie to pamiętam, choć nie staram się sobie tego przypominać. Po prostu przypomina mi się to | 0 | 1 | 2 | 3 | 4 |
| 9. Niektóre emocje, nastroje albo myśli przywołują wspomnienia przeszłych wydarzeń – choć nie próbuję ich sobie przypominać celowo | 0 | 1 | 2 | 3 | 4 |
| 10. Gdy jestem aktywna/ny fizycznie, na przykład podczas spacerowania, jazdy na rowerze, biegania, wyobrażenia dotyczące przyszłych wydarzeń same przychodzą mi na myśl – choć nie próbuję ich sobie przypominać celowo | 0 | 1 | 2 | 3 | 4 |

**Wersja Oryginalna:** Berntsen, D., Rubin, D. C., & Salgado, S. (2015). The frequency of involuntary autobiographical memories and future thoughts in relation to daydreaming, emotional distress, and age. *Consciousness and cognition, 36,* 352–372. https://doi.org/10.1016/j.concog.2015.07.007

**Adaptacja Polska:** Barzykowski, K., et al. (2026). Convergent validation of the Involuntary Autobiographical Memory Inventory across levels of analysis in a Polish sample. *Scientific Reports.* DOI: https://doi.org/10.1038/s41598-026-40606-3

| | | | | | |
|---|---|---|---|---|---|
| 11. Słuchanie muzyki lub pewnych utworów wywołuje wspomnienia przeszłych wydarzeń – choć nie próbuję ich sobie przypominać celowo | 0 | 1 | 2 | 3 | 4 |
| 12. Gdy dzieje się coś nieoczekiwanego, mimowolnie wyobrażam sobie podobne wydarzenia w przyszłości, choć nie staram się tego robić. Po prostu przychodzą mi na myśl | 0 | 1 | 2 | 3 | 4 |
| 13. Gdy odpoczywam lub wykonuję rutynowe czynności, wspomnienia przeszłych wydarzeń same przychodzą mi na myśl – choć nie próbuję ich sobie przypominać celowo | 0 | 1 | 2 | 3 | 4 |
| 14. Gdy się nudzę, wspomnienia przeszłych wydarzeń same przychodzą mi na myśl – choć nie próbuję ich sobie przypominać | 0 | 1 | 2 | 3 | 4 |
| 15. Pewne wrażenia sensoryczne, na przykład określone zapachy lub smaki, wywołują we mnie wyobrażenia przyszłych wydarzeń – choć nie próbuję ich sobie przywoływać celowo | 0 | 1 | 2 | 3 | 4 |
| 16. Po tym, jak doświadczyłam/łem coś, co zrobiło na mnie duże wrażenie, mimowolnie wyobrażam sobie podobne wydarzenia w przyszłości, choć nie staram się tego robić. Po prostu przychodzą mi na myśl | 0 | 1 | 2 | 3 | 4 |
| 17. Gdy jestem aktywna/ny fizycznie, na przykład podczas spacerowania, jazdy na rowerze, biegania, wspomnienia przeszłych wydarzeń same przychodzą mi na myśl – choć nie próbuję ich sobie przypominać celowo | 0 | 1 | 2 | 3 | 4 |
| 18. Słuchanie muzyki lub pewnych utworów wywołuje we mnie wyobrażenia przyszłych wydarzeń – choć nie próbuję ich sobie przywoływać celowo | 0 | 1 | 2 | 3 | 4 |
| 19. Niektóre miejsca i lokalizacje wywołuje we mnie wspomnienia przeszłych wydarzeń – choć nie próbuję ich sobie przypominać celowo | 0 | 1 | 2 | 3 | 4 |
| 20. Pewne wrażenia sensoryczne, na przykład określone zapachy lub smaki, wywołują we mnie wspomnienia przeszłych wydarzeń – choć nie próbuję ich sobie przypominać celowo | 0 | 1 | 2 | 3 | 4 |

**Wersja Oryginalna:** Berntsen, D., Rubin, D. C., & Salgado, S. (2015). The frequency of involuntary autobiographical memories and future thoughts in relation to daydreaming, emotional distress, and age. *Consciousness and cognition, 36,* 352–372. https://doi.org/10.1016/j.concog.2015.07.007

**Adaptacja Polska:** Barzykowski, K., et al. (2026). Convergent validation of the Involuntary Autobiographical Memory Inventory across levels of analysis in a Polish sample. *Scientific Reports.* DOI: https://doi.org/10.1038/s41598-026-40606-3
